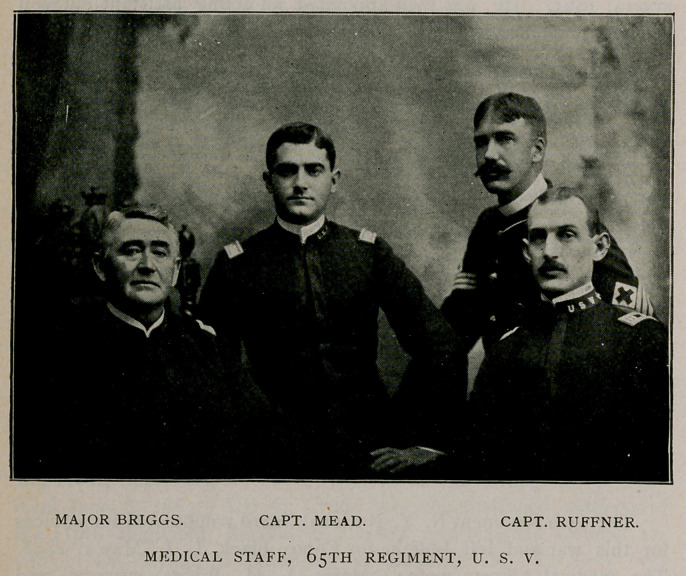# A Century of Medical History in the County of Erie.—1800–1900

**Published:** 1899-03

**Authors:** William Warren Potter

**Affiliations:** Buffalo, N. Y.


### A CENTURY OF MEDICAL HISTORY IN THE COUNTY OF ERIE.—1800–1900.

By WILLIAM WARREN POTTER, M. D., Buffalo, N. Y.

*Pioneer Physicians—Medical Societies—Medical Colleges—Hospitals—Medical Journals—Women Physicians—History of Homeopathy—Medical Officers of the Civil War—Individual Members of the Profession.*

[*Continued from the February edition.*]

### VII.—Medical Officers in the Civil War and the War with Spain.

Buffalo and Erie county contributed liberally to the medical staff of the army and navy during the civil war.

Dr. Charles H. Wilcox was commissioned surgeon of the 21st Regiment, May 15, 1861. He was the first physician to offer his services from Erie county, and was one of the ablest medical officers that went into the field from this region. He served until November 7, 1862, when he died of a disease contracted in the field with the Army of the Potomac.

Dr. Joseph A. Peters, son of the Hon. T. C. Peters, of Darien, Genesee county, was commissioned assistant surgeon of the 21st Regiment, May 15, 1861. He was appointed surgeon of the 6th Regiment, New York Cavalry, November 7, 1862. He retired from service before the regiment's time expired, and returned to Buffalo, where he engaged in practice. He afterward removed to the west and died at St. Louis.

Lucien Damainville, a student of Professor Frank Hastings Hamilton, was appointed assistant surgeon in the 31st New York Regiment at its organisation. He afterward became surgeon of the same regiment. He died at New York City, December 15, 1891.

Aaron J. Steele was commissioned assistant surgeon of the 26th New York Regiment, and soon after the expiration of his term of service located at St. Louis, where he now resides, and is engaged in teaching and practising orthopedic surgery.

Charles K. Winne, son of Dr. Charles Winne, was commissioned assistant surgeon in the United States Army in 1861, and is now a surgeon in the army still in active service.

Samuel D. Flagg was appointed assistant surgeon U. S. Navy in 1861.

Newton L. Bates was commissioned assistant surgeon United States Navy in 1861, and finally rose to the rank of Surgeon-General. He died while holding this office at Washington, D. C., October 18, 1898.

Ira C. Whitehead was appointed a surgeon in the Revenue Cutter service in 1861, and assigned to duty on board the "Vixen."

William Warren Potter was commissioned assistant surgeon of the 49th New York Regiment, September 16, 1861; surgeon of the 57th New York Regiment, December 16, 1862; served with the Army of the Potomac in the field until the close of the war, when he was brevetted Lieutenant-colonel of the United States Volunteers for "faithful and meritorious service." He is now engaged in practice at Buffalo and is editor of the BUFFALO MEDICAL JOURNAL.

CHARLES H. WILCOX, M. D.,

Surgeon 21st N. Y. Volunteers.

Sanford B. Hunt was appointed surgeon United States Volunteers in 1862, was assigned to the charge of convalescent camp near Alexandra, Va., in 1863, was an active surgeon in the field and at the end of the war compiled a military history of the United States sanitary commission. He died April 26, 1884, and was buried at Forest Lawn, Buffalo.

Albert J. Meyer was commissioned assistant surgeon United States army in 1853, invented a code of military signals, and was placed at the head of the signal bureau with the rank of Colonel in 1862; Brevet-Brigadier General at the end of the war, and later established the weather signal service. He died August 24, 1880, and his remains were interred at Forest Lawn, Buffalo.

E. P. Gray was appointed surgeon of the 100th New York Regiment, but did not take the field with that command. He afterward served as surgeon of the 78th New York Regiment, to which he was appointed February 11, 1862, and was discharged from service September 30, 1864. He died at St. Joseph, Mo., August 9, 1872.

Elias L. Bissell was commissioned assistant surgeon of the 44th Regiment, August 29, 1861, and served with the regiment in the field until November 20, 1862, when he was discharged. He was commissioned as surgeon of the 22d New York Infantry, December 5, 1862, and mustered out with his regiment June 19, 1863. He is now engaged in medical practice in Buffalo.

James W. Casey was commissioned assistant surgeon of the 105th New York Regiment, April 10, 1862, and was mustered out upon consolidation of the regiment, March 17, 1863.

William H. Butler served first as assistant surgeon of the 16th Regiment Michigan Volunteers, and afterward as acting assistant surgeon United States Army. In the latter capacity he was assigned to duty at Armory Square hospital, Washington, D. C., where he died February 5, 1864.

Sylvester Rankin was commissioned assistant surgeon in New Mexico Volunteers early in the war, but we have been unable to trace his service.

Heman P. Babcock was appointed assistant surgeon United States navy, and after the war was obliged to reside in California on account of his health. He died at Buffalo, December 27, 1877.

Chancey B. Hutchins was commissioned surgeon of the 116th Regiment, September 8, 1862, and was mustered out with his regiment June 8, 1865. Uri C. Lynde was commissioned surgeon of the 116th Regiment, September 8, 1862, and resigned October 12, 1863. Dr. Lynde is practising at Buffalo. M. E. Shaw was commissioned assistant surgeon of the 89th New York Infantry, December 10, 1862, and resigned October 11, 1863; appointed assistant surgeon of the 116th Regiment, March 12, 1864, and was mustered out of service with his regiment June 8, 1865. He was appointed an assistant surgeon United States Army and died October 11, 1867, on his way to join his command.

William D. Murray was commissioned assistant surgeon of the 100th Regiment, February 7, 1862, and discharged July 6, 1864. He located at Tonawanda, where he continued in practice for many years.

Frank Hastings Hamilton was commissioned surgeon of the 31st Regiment, May 25, 1861; was appointed surgeon United States Volunteers, September 1, 1861, and afterward was assigned to duty as medical director of the 4th Army Corps. After the war he taught and practised surgery in New York until his death, August 11, 1886.

William H. Gail in 1862 was appointed a medical cadet, United States Army and served as such in Stanton Hospital at Washington, D. C. He was commissioned assistant surgeon in the 18th Regiment, New York Cavalry, February 3, 1864, and resigned February 14, 1865. He was then appointed an acting assistant surgeon in the United States Army. He is now in active practice at East Aurora.

Carey W. Howe was commissioned assistant surgeon of the 116th New York Regiment, September, 1862, and resigned January 6, 1863. He is engaged in the practice of medicine at Buffalo.

Nehemiah Osborne served in a medical capacity during the civil war, but we have been unable to ascertain its nature. He died at Buffalo,———, 1896.

Justin G. Thompson was commissioned surgeon of the 77th Regiment, December 16, 1864, and was mustered out with his command June 27, 1865. He is now practising medicine at Angola.

S. S. Greene served as assistant surgeon in the United States Navy during a portion of the war, but in 1875 located at Buffalo, where he is still engaged in the practice of his profession.

### The American-Spanish War.

The 65th Regiment N. Y. National Guard volunteered its services for this war and left Buffalo for Camp Black, L. I., May 1, 1898. The medical officers were: Major A. H. Briggs, surgeon, and Captains Harry Mead and E. L. Ruffner, assistant surgeons. These officers were mustered into the service of the United States at Camp Black, and on May 18th the regiment proceeded to Camp Alger, Va. It remained at this station until September 5, 1898, when it returned to Buffalo and a month later was mustered out of the service. It is proper to add that two Buffalo physicians were commissioned as battalion majors in the 65th—Drs. Eugene A. Smith and J. D. Howland.

All these physicians returned to Buffalo with the regiment, serving continuously from the beginning until the end of the war.

During the summer of 1898, the 202d Regiment of Infantry, New York Volunteers, was organised at Buffalo, in which Dr.

Marshall Clinton was commissioned one of the assistant surgeons. This regiment is at the present writing still in service and is doing duty in the province of Pinar del Rio, Cuba.

In the early part of the war Dr. Francis T. Metcalf and C. Frank Bruso were appointed brigade surgeons. The former served on the hospital ship "Relief" and the latter was assigned to duty at Chicka-

O. O. HICKS, H. C.

MAJOR BRIGGS. CAPT. MEAD. CAPT. RUFFNER.

MEDICAL STAFF, 65TH REGIMENT, U. S. V.

mauga. Both have been mustered out, their services being no longer required.

The following-named physicians entered the service fiom Buffalo as acting assistant surgeons, to-wit: Edward J. Meyer, Ira C. Brown, Francis T. Metcalfe, John E. Bacon, Nelson W. Wilson, George W. Pattison, H. H. Bradley, John H. Grant, Vertner Kennerson, Julius Ullman, J. N. Goltra, Harold W. Cowper, Charles W. Farr, W. T. Tanner, Charles A. Andrews. Drs. Brown and Metcalfe were subsequently promoted to be brigade surgeons.

(*Continued next month.*)

## Figures and Tables

**Figure f1:**
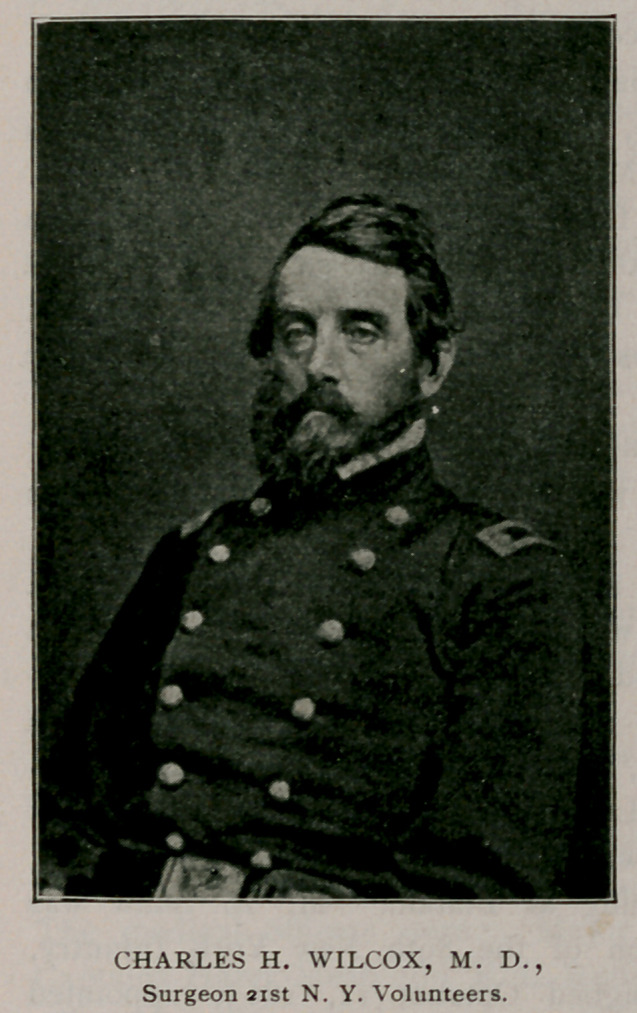


**Figure f2:**